# Meta-Analysis of 11 Heterogeneous Studies regarding Dipeptidyl Peptidase 4 Inhibitor Add-On Therapy for Type 2 Diabetes Mellitus Patients Treated with Insulin

**DOI:** 10.1155/2020/6321826

**Published:** 2020-11-11

**Authors:** Katsuya Shibuki, Shuji Shimada, Takao Aoyama

**Affiliations:** ^1^Department of Pharmacy, Tokyo University of Science, 2641 Yamazaki, Noda 278-8510, Japan; ^2^Clinical Research Center, Medical Hospital, Tokyo Medical and Dental University, 1-5-45 Yushima, Tokyo 113-8519, Japan

## Abstract

**Background:**

Several clinical trials have addressed the therapeutic strategy of adding dipeptidyl peptidase 4 (DPP-4) inhibitors to the treatment of type 2 diabetes mellitus (DM) inadequately controlled by insulin therapy. However, there is a high degree of heterogeneity in these studies, and the cause of which has not been identified.

**Methods:**

We conducted a meta-analysis of randomized controlled trials, which compared the efficacy and safety of adding DPP-4 inhibitors or placebo to insulin therapy; the level of hemoglobin A1c (HbA1c) in the patients was >7.0%, and the duration of treatment was ≥8 weeks. We focused on the mean changes in HbA1c from the baseline (*Δ*HbA1c) and the incidence of hypoglycemia. We assumed that five baseline parameters (HbA1c, fasting blood glucose, body mass index (BMI), duration of type 2 DM, and duration of treatment) could affect *Δ*HbA1c. Regarding the incidence of hypoglycemia, we suspected that the heterogeneity was caused by differences in the definition of hypoglycemia among the studies.

**Results:**

Data obtained from 11 studies (*n* = 4654 patients) were included in the analysis. The mean *Δ*HbA1c between the DPP-4 inhibitor and placebo groups was -0.61% (95% confidence interval (CI): -0.74 to -0.48, *I*^2^ = 73.4%). There was substantial heterogeneity among the 11 studies, but 74.1% of this variability was explained by the difference in BMI. The odds ratio for the incidence of hypoglycemia was 1.02 (95% CI: 0.74 to 1.42, *I*^2^ = 63.8%), with substantial heterogeneity due to differences in the definition of hypoglycemia among the studies. There was no apparent effect of publication bias.

**Conclusions:**

The addition of DPP-4 inhibitors to insulin therapy for adult patients with type 2 DM can significantly reduce HbA1c levels without increasing the occurrence of hypoglycemia. BMI and hypoglycemia definition could explain the heterogeneity in the clinical trials. This trial is registered with PROSPERO #CRD42016035994.

## 1. Introduction

The control of blood glucose level is important for preventing microvascular-related complications associated with type 2 diabetes mellitus (DM) treatment [1–5]. Eventually, many patients with type 2 DM require insulin therapy. However, a meta-analysis of randomized controlled trials reported that 50%–65% of patients receiving insulin therapy failed to achieve their glycemic control goals [6].

Dipeptidyl peptidase 4 (DPP-4) inhibitors are oral hypoglycemic agents that prevent the inactivation of glucagon-like peptide 1 (GLP-1) and gastric inhibitory polypeptide (GIP) and enhance the effects of endogenous incretin [7–10]. GLP-1 acts on *β* cells and increases insulin secretion in a glucose-dependent manner. In addition, GLP-1 exerts a glucose-dependent inhibitory effect on glucagon secretion. Because of these effects, DPP-4 inhibitors have a low risk of hypoglycemic incidence, and they are suitable as an add-on therapy with insulin.

Several clinical trials have addressed the therapeutic strategy of adding DPP-4 inhibitors to the treatment of type 2 DM inadequately controlled by insulin therapy. In 2015, Chen et al. performed a meta-analysis to combine seven clinical studies and showed that DPP-4 inhibitor therapy has a low risk of hypoglycemia and that it improves glycemic control [11]. Subsequently, several investigators confirmed these conclusions using a meta-analysis approach on the same topic [12–14]. However, there is a high degree of heterogeneity in these meta-analyses, possibly because of variations in the designs and patient backgrounds among the clinical trials, and the cause of this variability has not been identified.

This high degree of heterogeneity suggests that the efficacy of DPP-4 inhibitors differs in each population, used in individual trials. If this assumption is true, it is inappropriate to combine populations with different drug efficacies using a standard meta-analysis approach. However, if the source of this heterogeneity is attributable to variations in other parameters (covariates), which characterize the population in each study, then it is reasonable to combine the data from individual trials using a meta-analysis approach, taking into account the effects of the covariates.

Thus, the aim of the present study was to identify covariates that could explain the observed variations among the clinical trials. Initially, we selected several baseline parameters that could affect the efficacy of DPP-4 inhibitors, namely, hemoglobin A1c (HbA1c), fasting plasma glucose (FPG), body mass index (BMI), type 2 DM duration, and treatment duration [15, 16]. We also selected the incidence of hypoglycemia as a safety index for DPP-4 inhibitors. There are two definitions of hypoglycemia, and the incidence of hypoglycemia could be affected by the definition chosen [17]. Therefore, we also tested the possibility that the differences in the definition of hypoglycemia can explain the heterogeneity in the incidence of hypoglycemia among the studies.

## 2. Materials and Methods

This review was conducted and reported according to the Preferred Reporting Items for Systematic Reviews and Meta-Analyses (PRISMA) [18]. The protocol was based on Preferred Reporting Items for Systematic Reviews and Meta-Analyses for Protocols 2015 (PRISMA-P 2015) [19] and was registered in the International Prospective Register of Systematic Reviews (PROSPERO, ID = CRD42016035994) [20].

### 2.1. Information Sources and Literature Search

In accordance with Peer Review of Electronic Search Strategies (PRESS) [21], we used MEDLINE, EMBASE, the Cochrane Library, ClinicalTrials.gov, and pharmaceutical company sites as sources of information. A comprehensive literature search was conducted from September 1, 2015, to December 31, 2016. The search keywords used were as follows: “dipeptidyl peptidase 4 inhibitors,” “sitagliptin,” “alogliptin,” “vildagliptin,” “saxagliptin,” “linagliptin,” “anagliptin,” “teneligliptin,” “insulin,” “lispro,” “aspart,” “glulisine,” “neural,” “isophane,” “glargine,” “detemir,” and “degludec.” There were no language restrictions in the search. In addition, a combination of related literature searches using PubMed and Mendeley, prospective citation searches using Google Scholar and the Web of Science™, and reviews of the included clinical studies and reviews related to the subject was investigated.

### 2.2. Inclusion and Exclusion Criteria

Patients with type 2 DM (≥18 years old, HbA1c ≥ 7.0%, excluding pregnant women), who had been treated with a fixed dose of insulin (insulin lispro, insulin aspart, insulin glulisine, insulin neural, insulin isophane, insulin glargine, insulin detemir, or insulin degludec; single agent or in combination with metformin) for more than 8 weeks before DPP-4 treatment, were enrolled. Randomized controlled trials of ≥12 weeks were selected for the analysis. The intervention group had received an additional DPP-4 inhibitor (sitagliptin, alogliptin, vildagliptin, saxagliptin, linagliptin, anagliptin, or teneligliptin) at the standard dosage coadministered with the insulin therapy. The control group had received placebo instead of a DPP-4 inhibitor with the insulin therapy.

### 2.3. Study Selection and Data Extraction

Two investigators (K.S. and S.S.) reviewed the titles and summaries of 3003 publications to determine whether they should be included in our analysis. The study design, subject data, and evaluated points in each study were summarized, and the bias in the study was assessed using “the Cochrane risk of bias tool” [22]. The two investigators discussed and resolved any discrepancies between them, and a consensus was reached.

### 2.4. Data Analysis

We evaluated the mean change in HbA1c (*Δ*HbA1c) and the incidence of hypoglycemia. First, we performed a meta-analysis using a random-effects model [23]. A random-effects model is a statistical model that assumes random variations among studies. We used the restricted maximum likelihood method to estimate *τ*, the index of variation between studies [24], and the Hartung–Knapp adjustment to calculate the confidence interval [25, 26]. We calculated the difference in *Δ*HbA1c between the intervention and control groups and the odds ratio for the incidence of hypoglycemia, with 95% confidence intervals for each. If no standard deviation for outcome was reported in a study, the standard error was calculated from the reported sample size and the 95% confidence interval.

We defined a full analysis set as all subjects who received a DPP-4 inhibitor or placebo at least once after randomization and whose data were available. In addition, we defined a safety analysis set as all subjects who received a DPP-4 inhibitor or placebo at least once after randomization. We analyzed mean *Δ*HbA1c based on the full analysis set data and the incidence of hypoglycemia based on the safety analysis set data. We collected data that were available in published manuscripts or on websites of pharmaceutical companies and trial registries. Additionally, if the data were not present in the results, we contacted the corresponding authors or sponsors for the data. When multiple reports were found in a study, we selected the report with the longest treatment duration. We assessed statistical heterogeneity with *I*^2^ [27]. In general, *I*^2^ values of 30%–60% and ≥75% were defined as moderate heterogeneity and considerable heterogeneity, respectively [22]. As a more stringent criterion, when heterogeneity was >50%, we determined that substantial heterogeneity existed and examined the causes by prespecified covariates and stratification.

Next, we performed a random-effects metaregression using a mixed-effects model [28]. A mixed-effects model is a statistical model that assumes the existence of variables (covariates) that can explain variability among studies. We assumed baseline HbA1C, FPG, BMI, type 2 DM duration at the beginning of each study, and treatment duration in each study as the candidates of such covariates. We evaluated the predictive value of the mixed-effects model with one or two of the covariates using Akaike's information criterion with a correction for small sample sizes (corrected AIC) [29, 30]. The studies were divided into the following two subgroups based on the incidence of hypoglycemia: those in which hypoglycemia was diagnosed based on certain criteria and those in which hypoglycemia was diagnosed as a symptom [17]. The risk of publication bias and other biases was assessed using contour-enhanced funnel plots [31]. All statistical analyses were performed using R version 3.3.0 (Copyright 2016 The R Foundation for Statistical Computing) and “metafor” (version 1.9-8) package [32].

## 3. Results

We retrieved 3003 publications based on internet searches, and finally, 11 studies were selected for the present analysis. The characteristics of these 11 studies are summarized in Table 1, the completed PRISMA checklist is described in Table S1, the search strategy is described in Table S2, and the flowchart for selecting the studies is described in Figure S1. The risks of bias assessments of the studies are summarized in Figures S2 and S3. There were a few risks of bias that would affect the assessment of efficacy and safety of DPP-4 inhibitors.

### 3.1. HbA1c Levels

**Figure 1:**
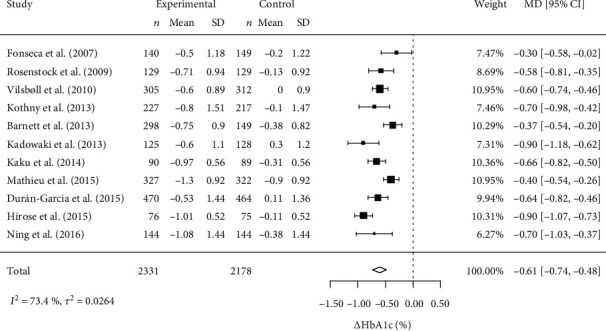
Forest plot for *Δ*HbA1c in the random-effects model. Each square indicates the mean difference (MD) and the bar indicates the 95% confidence interval (CI) from an eligible study. The size of each square corresponds to the weight of that study. The diamond and its width represent the combined MD and 95% CI, respectively. *Δ*HbA1c: change in hemoglobin A1c; SD: standard deviation.

For *Δ*HbA1c, we first performed a meta-analysis using the random-effects model. The results are shown in Figure 1 using a forest plot.

The *Δ*HbA1c was -0.61% (95% CI: -0.74 to -0.48, 4509 patients from 11 studies), indicating that the addition of a DPP-4 inhibitor significantly reduced HbA1c levels compared with the placebo. There were no outliers that significantly affected the accuracy of the model (Figure S4). However, there was substantial heterogeneity among the 11 studies, with *I*^2^ = 73.4%. These results suggest that the efficacy of the DPP-4 inhibitors differs among the 11 studies.

We attempted to explain this heterogeneity by performing a random-effects metaregression using a mixed-effects model. As a reference to select the most appropriate model, we calculated two statistical parameters, AIC and pseudo *R*^2^ statistics [44]. The AIC shows the predictability of a statistical model. The smaller the AIC, the better the model's prediction. Pseudo *R*^2^ is the percentage of total heterogeneity explained by covariates. The larger the pseudo *R*^2^, the more sufficiently the heterogeneity can be explained. Due to the limited number of studies used in the present analysis, we limited the number of input covariates to one or two to prevent overfitting, and we made a finite correction to AIC (i.e., corrected AIC) [30]. The calculation results are shown in Table 2.

BMI accounted for 74.1% of the heterogeneity (pseudo *R*^2^ = 74.1%, test of covariates: *p* = 0.0036). A diagnostic plot indicated that the study of Kaku et al. may have reduced the accuracy of the prediction (Figure S5). However, the model with a covariate of BMI had the best predictive performance even with the 10 studies excluding that of Kaku et al. Therefore, we concluded that it is reasonable to select BMI as the sole covariate that explains the heterogeneity in *Δ*HbA1c.

**Figure 2:**
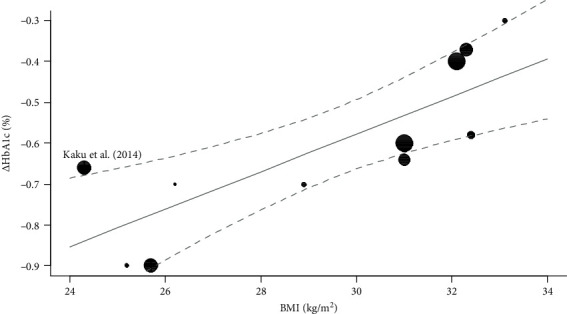
Bubble plot showing the relationship between BMI and *Δ*HbA1c. The vertical axis represents *Δ*HbA1c in each study, and the horizontal axis shows the corresponding BMI. The diameter of the circles reflects the weight of each study in the combined results. Gray solid lines indicate the regression line, and dotted lines indicate the 95% confidence interval of the regression line. BMI: body mass index; *Δ*HbA1c: change in hemoglobin A1c.

The relationship between BMI and *Δ*HbA1c is shown in Figure 2 using a bubble plot. From Figure 2, it can be concluded that *Δ*HbA1c increases with an increase in BMI.

The relationship between HbA1c and BMI can be expressed by the following formula.

(1) $$\begin{array}{c} \Delta \text{HbA}1\text{c}=0.0459*\text{BMI}-1.9546. \end{array}$$

According to this formula, we adjusted the *Δ*HbA1c according to the BMI. We shifted the *Δ*HbA1c in each study by the following amount (equation (2)), whereas the variance in the study was unchanged.

(2) $$\begin{array}{c} \begin{array}{c} 0.0459*\left(\text{mean}\text{BMI}\text{in}\text{the}11\text{studies}-\text{mean}\text{BMI}\text{in}\text{the}\text{particular}\text{study}\right). \end{array} \end{array}$$

**Figure 3:**
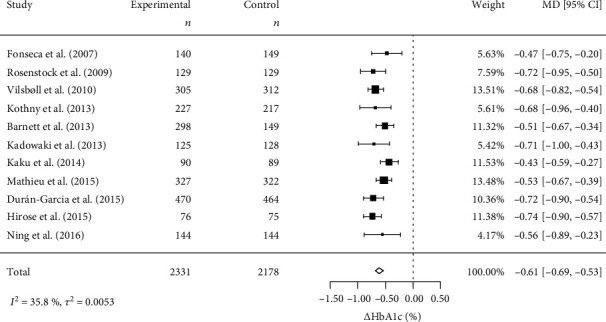
Forest plot for corrected *Δ*HbA1c based on the BMI in each study. BMI: body mass index; *Δ*HbA1c: change in hemoglobin A1c; MD: mean difference; CI: confidence interval.

The results are shown in Figure 3 using a forest plot. This manipulation clearly reduced the overall variability among the studies and improved the accuracy of integration.

**Figure 4:**
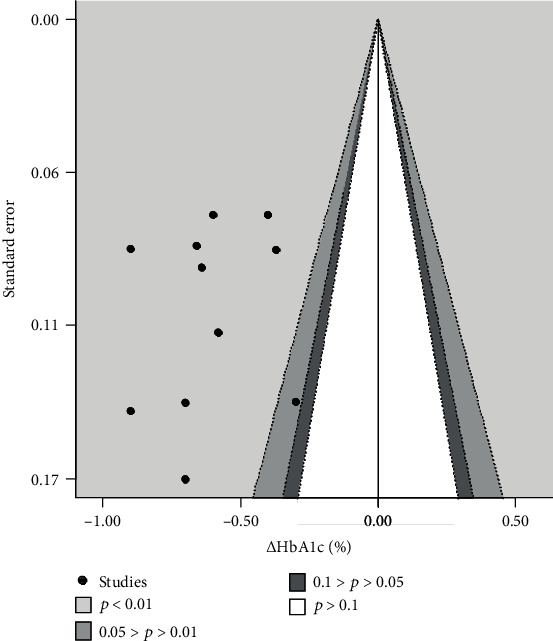
Contour-enhanced funnel plot for *Δ*HbA1c. The vertical axis represents the standard error in each study, and the horizontal axis represents the *Δ*HbA1c. The studies in the colored area are those in which *Δ*HbA1c significantly deviated from zero with DPP-4 inhibitors. *Δ*HbA1c: change in hemoglobin A1c; DPP-4: dipeptidyl peptidase 4.

The effects of bias are shown in Figure 4 using a contour-enhanced funnel plot.

### 3.2. Hypoglycemic Incidence

**Figure 5:**
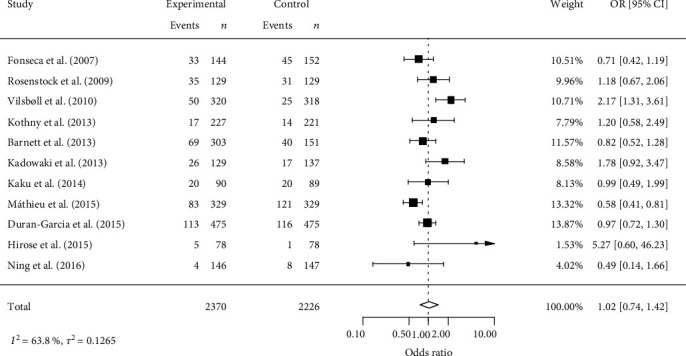
Forest plot for hypoglycemic incidence in the random-effects model. Each square indicates the odds ratio (OR) and the bar indicates the 95% confidence interval (CI) from an eligible study. The size of each square corresponds to the weight of that study. The diamond and its width represent the combined OR and 95% CI, respectively.

For hypoglycemic incidence, we performed a meta-analysis using the random-effects model. The results are shown in Figure 5 using a forest plot.

The odds ratio of the hypoglycemic incidence rate was 1.02 (95% CI: 0.74 to 1.42, 4596 patients from 11 studies), indicating that adding a DPP-4 inhibitor did not significantly increase the incidence of hypoglycemia. However, there was an outlier that significantly affected the accuracy of the model, and there was substantial heterogeneity among the 11 studies, with *I*^2^ = 63.8%.

**Figure 6:**
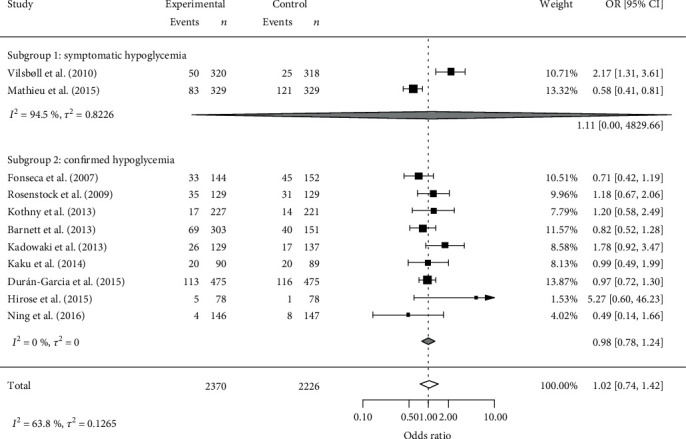
Forest plot for hypoglycemic incidence in the two groups of studies stratified based on the definition of hypoglycemic incidence. OR: odds ratio; CI: confidence interval.

A diagnostic plot indicated that the study of Vilsbøll et al. may have reduced the accuracy of the prediction (Figure S6). In that study, hypoglycemic incidence diagnosed based only on clinical symptoms and blood glucose level was not used as a criterion. The study by Mathieu et al., which also adopted only clinical symptoms as the criteria for hypoglycemic incidence, may also have reduced the prediction accuracy of the random-effects model. In the other nine studies, hypoglycemic incidence was confirmed based on clinical symptoms and laboratory measurements of blood glucose levels. These results suggest that the definition of hypoglycemia is responsible for the heterogeneity in the OR of the hypoglycemic incidence rate. Thus, we divided the 11 studies into the following two subgroups: subgroup 1 with symptomatic hypoglycemia and subgroup 2 with confirmed hypoglycemia. The results of our stratification are shown in Figure 6.

The OR for hypoglycemic incidence rate was 1.11 (95% CI: 0–4829.66) in subgroup 1 and 0.98 (0.78 to 1.24) in subgroup 2. Subgroup 1 showed a high degree of heterogeneity with *I*^2^ = 94.5%, whereas the heterogeneity in subgroup 2 was eliminated (*I*^2^ = 0%). Thus, our stratification based on the definition of hypoglycemia used in each study explained the heterogeneity in the hypoglycemic incidence rate.

**Figure 7:**
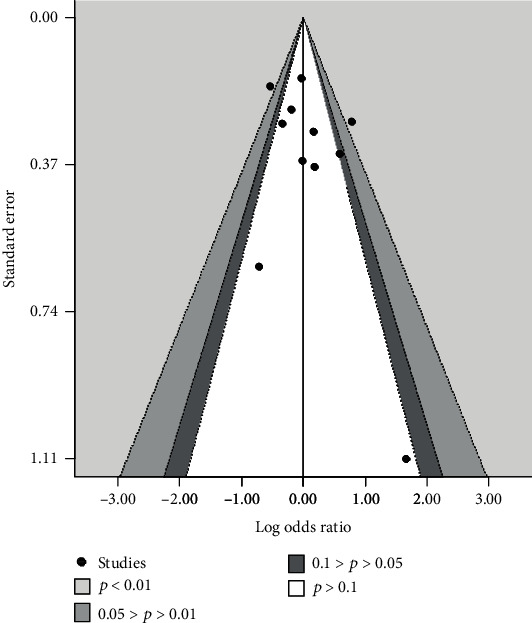
Contour-enhanced funnel plot for hypoglycemic incidence. The vertical axis represents standard error in each study, and the horizontal axis shows log odds ratio for the incidence of hypoglycemia. The studies in the colored area are those in which the incidence of hypoglycemia was significantly affected by DPP-4 inhibitors. DPP-4: dipeptidyl peptidase 4.

The effects of bias are shown in Figure 7 using a contour-enhanced funnel plot.

## 4. Discussion

DPP-4 inhibitors are oral antidiabetic agents, which are usually administered before insulin therapy that requires subcutaneous injections [45, 46]. In contrast, in the present study, we examined the efficacy and safety of a specific treatment strategy of administering a DPP-4 inhibitor to patients who have already received insulin therapy. Thus, only 11 studies were available for our meta-analysis. All existing meta-analyses were consistent in their conclusions that DPP-4 inhibitors significantly reduced HbA1c levels without increasing hypoglycemia (Table 3). However, all meta-analyses also showed substantial heterogeneity in either HbA1c, hypoglycemia, or both, and the reason underlying this variability was not clear [11–14].

Without a rational explanation for the heterogeneity, the predictive performance of statistical models is unreliable, and thus, the safety and efficacy of DPP-4 inhibitors as add-on therapies cannot be assured. In this study, we examined various covariates using mixed-effects models and found that the BMI could explain most of the heterogeneity.

### 4.1. Effect of DPP-4 Inhibitors on the *Δ*HbA1c

First, we performed a meta-analysis using the random-effects model without covariates. The *Δ*HbA1c was -0.61% (95% CI: -0.74 to -0.48), indicating that adding a DPP-4 inhibitor significantly reduced HbA1c levels compared with that of the placebo. However, there was substantial variability among the 11 studies (*I*^2^ = 73.4%). We then examined five covariates, which we had selected in advance using the mixed-effects models, and found that a sole covariate, BMI, accounted for 74.1% of the heterogeneity.

The BMI, with the duration of type 2 DM as a second covariate, explained 88.5% of the heterogeneity, but the predictive performance was significantly reduced. Other combinations of covariates were possible. However, many covariates would easily lead to overfitting and less predictive power of the data because the number of studies was limited. Therefore, we concluded that it is reasonable to select BMI as the sole covariate that explains the heterogeneity in the *Δ*HbA1c.

Home et al. reported in an observational study that the association between *Δ*HbA1c and BMI was barely significant in 21854 insulin-treated patients. In their study, the coefficient of determination was 0.03 and the regression equation for BMI could hardly explain the actual data [16]. However, observational studies have limitations in the evaluation of covariates because of unpredictable confounders and various therapeutic interventions that depend on metabolic factors, including BMI. As we targeted a specific treatment strategy of administering a DPP-4 inhibitor to patients with type 2 DM who were nonresponsive to insulin, the covariates affecting the *Δ*HbA1c may be different compared with those in the study of Home et al. Our meta-analysis included only randomized controlled trials with limited interventions, and the effect of confounders was considered minimal. Contrary to the findings of Home et al., our meta-analysis of randomized controlled trials does suggest that BMI influences the efficacy of DPP-4 inhibitors.

Kim et al. reported that the efficacy of DPP-4 inhibitors in type 2 DM treatment was higher in Asians than in other ethnic groups, and the percentage of Asian participants correlated with the average BMI [47]. One explanation for the relationship between BMI and *Δ*HbA1c is that racial differences may be associated with both BMI and *Δ*HbA1c. Among the 11 studies analyzed in our meta-analysis, four were conducted in Asian countries (China, Japan, Philippines, Singapore, and Thailand) with a baseline mean BMI of <27. As the proportion of Asians is considerably lower in the other seven studies, there might be an association between the BMI and racial differences. It should be noted that Asians are genetically diverse, and therefore, it is difficult to prove a relationship between racial differences and BMI. Accordingly, it has been reported that Asians have diverse responses to DPP-4 inhibitors [48–50]. A meta-analysis of randomized controlled trials in racially homogeneous patients with type 2 DM who have already received insulin therapy may be useful in testing the pure effect of BMI on the efficacy of DPP-4 inhibitors without the influence of ethnic backgrounds. However, there are only a small number of such studies available for a meta-analysis.

Another explanation for the relationship between BMI and *Δ*HbA1c is that adiponectin may be associated with both BMI and *Δ*HbA1c. Adiponectin is a cytokine secreted from adipose tissue [51]. A positive correlation has been observed between insulin sensitivity and adiponectin levels both *in vitro* and *in vivo* [52, 53]; thus, decreased blood adiponectin levels should be associated with increased insulin resistance [54, 55]. Because blood adiponectin levels are inversely correlated with BMI, patients with high BMIs may not have responded to insulin therapy and to adjuvant DPP-4 inhibitors that facilitate the effects of insulin. It has also been suggested that differences in adiponectin levels are responsible for the variability in the effects of DPP-4 inhibitors in Asians [50]. However, it is difficult to identify the responsible metabolic factors underlying the relationship between BMI and *Δ*HbA1c; it could be adiponectin, other factors related to BMI, or factors related to ethnic backgrounds, but it may not be related directly to BMI.

### 4.2. Effect of DPP-4 Inhibitors on Hypoglycemic Incidence

First, we performed a meta-analysis using the random-effects model without covariates. The odds ratio for hypoglycemic incidences was 1.02 (95% CI: 0.74–1.02), indicating that adding a DPP-4 inhibitor did not significantly increase hypoglycemic incidence compared with that of the placebo. However, there was a substantial heterogeneity among the 11 studies (*I*^2^ = 63.8%). We reviewed the 11 studies used in the meta-analysis using diagnostic plots. As a result, we found that hypoglycemic incidences were diagnosed in the studies based on different criteria; in two studies, hypoglycemic incidences were diagnosed based only on clinical symptoms (subgroup 1), and in the remaining nine studies, hypoglycemic incidences were assessed by blood glucose levels in addition to clinical symptoms (subgroup 2). Therefore, we attempted to stratify the two types of studies based on the hypoglycemic diagnostic criteria before performing mixed-effects model analysis with the covariates. This stratification analysis is predefined.

In subgroup 1, the odds ratio for hypoglycemic incidences was 1.11 (0–4829.66, *I*^2^ = 94.5%). We judged that the informational value of the combined results was low because of the very wide CI. The two studies also demonstrated a high heterogeneity with different directions of effect, which suggests that defining hypoglycemia based solely on clinical symptoms is unreliable. However, in subgroup 2, the odds ratio for hypoglycemic incidences was 0.98 (0.78 to 1.24, *I*^2^ = 0%). All nine studies were consistent in that the hypoglycemic incidence was not significantly increased, and there was no heterogeneity. These results suggest that the objective definition of hypoglycemic incidence using blood glucose levels is reliable. It is concluded that DPP-4 inhibitors do not increase hypoglycemic incidence. Thus, even if the effect of a DPP-4 inhibitor is influenced by BMI, the former covariate unlikely has any apparent effect on the odds ratio of the hypoglycemic incidence.

### 4.3. Assessment of Publication Bias

The contour-enhanced funnel plot for *Δ*HbA1c appeared to be asymmetric (Figure 4). However, because the direction of the effect was consistent and all studies were plotted in the area that showed statistically significant decreases in HbA1c levels, the asymmetry in the contour-enhanced funnel plot was believed to be due to the consistent demonstration of treatment effect.

The contour-enhanced funnel plot for hypoglycemic incidence lacked data points in the lower left region (Figure 7). As this region represents DPP-4 inhibitors reducing the hypoglycemic incidence, the possibility of publication bias was low. In addition, even if there is a lack of research in this area due to bias, the influence would be small because the standard error is large and the weight on the combined results would be small.

In general, Egger's test is often used to check for publication bias [56], but because it does not have sufficient statistical power when the number of studies used in the analysis is small [57] and because increasing statistical hypothesis testing, such as with Egger's test, increases the risk of overall type I errors, we decided not to perform the test [58]. Moreover, we do not believe that running Egger's test would have a significant impact on the conclusions of this meta-analysis.

### 4.4. Strengths and Limitations of This Meta-Analysis

The strength of our study is that we have shown for the first time, using a random-effects metaregression approach, the source of heterogeneity between studies on the therapeutic strategy of adding DPP-4 inhibitors to insulin therapy. We resolved the concern of a high degree of heterogeneity in the evidence for the addition of DPP-4 inhibitors to treatment regimens for patients experiencing inadequate glycemic control with insulin, and the evidence is now strengthened.

The result that BMI can explain most of the heterogeneity between studies indicates that we may predict the HbA1c-lowering effects of DPP-4 inhibitors more accurately by considering BMI values in clinical practice. Although it is important to note that ethnic differences may exist and that there are cases of visceral obesity that cannot be assessed by BMI, we believe that considering BMI can help achieve treatment goals.

Regarding the result that the definition of hypoglycemia may be a source of heterogeneity, we would like to emphasize its impact on the design of future clinical studies rather than its clinical significance. The results of our analysis suggest that reporting the objective incidence of hypoglycemia, as confirmed by laboratory values, is more likely to determine the effect of the test drug than hypoglycemia as determined more accurately by symptoms alone.

On the other hand, the limitation of our study is that we were unable to perform a detailed analysis of the covariates because we used summary values. Although we established the covariates in advance based on the literature, the possibility of unknown covariates cannot be ruled out. An individual patient data (IPD) meta-analysis is ideal for advanced analyses that consider the search for unknown covariates. However, collecting sufficient data to conduct an IPD meta-analysis is difficult, to begin with [59], and considering the unpredictable impact of data availability bias, we conducted a standard meta-analysis of aggregate data.

## 5. Conclusions

In patients who do not respond to insulin therapy, the addition of a DPP-4 inhibitor can reduce HbA1c levels without increasing hypoglycemic incidence. As for *Δ*HbA1c, mixed-effects models with BMI as a single covariate explained the heterogeneity among studies and improved the accuracy of meta-analysis integration. Random-effects metaregression is a simple approach that can be applied to other meta-analyses and may be a useful tool that provides new insights into factors influencing drug effects along with improved accuracy and reliability of meta-analytic integration.

## Figures and Tables

**Table 1:** Characteristics of the 11 trials.

	Number of patients (experimental vs. control)	Country	Type 2 DM duration^a^ (years)	HbA1c (%)	FPG (mg/dL)	BMI (kg/m^2^)	Therapy duration (weeks)
Fonseca et al. [33]	296 (144/152)	Germany, Finland, Spain, USA	14.7	8.40	161.8	33.1	24
Rosenstock et al. [34]	259 (130/129)	13 countries	12.8	9.30	190.8	32.4	26
Vilsbøll et al. [35]	641 (322/319)	22 countries	12.0	8.60	177.1	31.0	24
Kothny et al. [36]	449 (228/221)	11 countries	13.0	8.80	NA	28.9	24
Barnett et al. [37]	455 (304/151)	11 countries	11.9	8.70	173.3	32.3	52
Kadowaki et al. [38]	266 (129/137)	Japan	14.0	8.90	165.0	25.2	16
Kaku et al. [39]	179 (90/89)	Japan	14.9	8.43	154.9	24.3	12
Mathieu et al. [40]	658 (329/329)	27 countries	13.4	8.80	176.4	32.1	24
Durán-Garcia et al. [41]	950 (475/475)	19 countries	NA	8.30	151.2	31.0	52
Hirose et al. [42]	156 (78/78)	Japan	12.9	8.10	160.2	25.7	12
Ning et al. [43]	293 (146/147)	China, Thailand, Philippines, Singapore	11.3	8.70	171.0	26.2	24

^a^Abbreviations: DM: diabetes mellitus; HbA1c: hemoglobin A1c; FPG: fasting plasma glucose; BMI: body mass index; NA: not available.

**Table 2:** Covariates for *Δ*HbA1c in the mixed-effects model.

Covariates	Corrected AIC^a^	Pseudo *R*^2^
BMI	-0.62	74.1
(Without covariate)	1.51	NA
Therapy in weeks	5.80	19.2
HbA1c	7.56	0
FPG	9.31	2.3
Duration of type 2 DM	10.43	0
BMI+HbA1c	11.96	67.9
BMI+therapy in weeks	12.16	66.5
Therapy in weeks+HbA1c	17.40	18.5
BMI+duration of type 2 DM	18.31	88.5
BMI+FPG	20.12	66.4
Therapy in weeks+duration of type 2 DM	21.47	62.4
Therapy in weeks+FPG	24.64	24.3
HbA1c+FPG	26.60	0
HbA1c+duration of type 2 DM	27.32	0
FPG+duration of type 2 DM	47.30	0

^a^Abbreviations: AIC: Akaike's information criterion; BMI: body mass index; HbA1c: hemoglobin A1c; FPG: fasting plasma glucose; DM: diabetes mellitus; NA: not applicable.

**Table 3:** Summary of the results of existing meta-analyses.

	*Δ*HbA1c (%)	Odds ratio for hypoglycemic incidence
Chen et al. [11]	-0.52% (-0.59 to -0.44, *I*^2^ = 0%, 7 studies)	1.04 (0.83 to 1.31, *I*^2^ = 58.5%, 7 studies)
Kim et al. [12]	-0.58% (-0.70 to -0.46, *I*^2^ = 76.4%, 9 studies)	0.94 (0.84 to 1.05, *I*^2^ = 71.7%, 9 studies)
Yang et al. [13]	-0.53% (-0.63 to -0.43, *I*^2^ = 99%, 7 studies)	1.02 (0.91 to 1.16, *I*^2^ = NR^a^, 7 studies)
Wang et al. [14]	-0.54% (-0.66 to -0.42, *I*^2^ = 82%, 22 studies)	0.92 (0.78 to 1.10, *I*^2^ = 60%, 22 studies)
Present study	-0.61% (-0.74 to -0.48, *I*^2^ = 73.4%, 11 studies)	1.02 (0.74 to 1.42, *I*^2^ = 63.8%, 11 studies)

^a^Abbreviation: NR: not reported.

## Data Availability

All datasets for this study are included in the tables and figures.

## Supplementary Materials

Supplementary Materials Table S1: completed PRISMA checklist. Table S2: search strategy. Figure S1: flow chart for selecting included studies. Figure S2: risk of bias assessment for *Δ*HbA1c. We evaluated “Other bias” as high risk when the placebo run-in period was not set. Figure S3: risk of bias assessment for the incidence of hypoglycemia. We evaluated “Blinding of outcome assessment” as high risk when symptomatic hypoglycemia was adopted as the definition of hypoglycemia. Figure S4: diagnostic plot of the random-effects model for *Δ*HbA1c. Figure S5: diagnostic plot of the mixed-effects model for *Δ*HbA1c. Study 7 is the study by Kaku et al. (2014). Figure S6: diagnostic plot of random-effects model for hypoglycemia. Study 3 and study 8 are the studies by Vilsbøll et al. (2010) and Mathieu et al. (2015), respectively.
