# Supplementary material for: Meta-Analysis of 11 Heterogeneous Studies regarding Dipeptidyl Peptidase 4 Inhibitor Add-On Therapy for Type 2 Diabetes Mellitus Patients Treated with Insulin
Source: J Diabetes Res. 2020 Nov 11;2020:6321826. doi: 10.1155/2020/6321826 (PMC7673952; doi:10.1155/2020/6321826)
Supplement: Supplementary Materials — Table S1: completed PRISMA checklist. Table S2: search strategy. Figure S1: flow chart for selecting included studies. Figure S2: risk of bias assessment for ΔHbA1c. We evaluated “Other bias” as high risk when the placebo run-in period was not set. Figure S3: risk of bias assessment for the incidence of hypoglycemia. We evaluated “Blinding of outcome assessment” as high risk when symptomatic hypoglycemia was adopted as the definition of hypoglycemia. Figure S4: diagnostic plot of the random-effects model for ΔHbA1c. Figure S5: diagnostic plot of the mixed-effects model for ΔHbA1c. Study 7 is the study by Kaku et al. (2014). Figure S6: diagnostic plot of random-effects model for hypoglycemia. Study 3 and study 8 are the studies by Vilsbøll et al. (2010) and Mathieu et al. (2015), respectively. [file 6321826.f1.docx]

## Supplementary Material

Table S1: Completed PRISMA checklist

| **Section/topic** | **ItemNo.** | **Checklist item** | **Reported on**  **page No.** |
| --- | --- | --- | --- |
| **TITLE** | | | |
| Title | 1 | Identify the report as a systematic review, meta-analysis, or both. | 1 |
| **ABSTRACT** | | | |
| Structured summary | 2 | Provide a structured summary including, as applicable: background; objectives; data sources; study eligibility criteria, participants, and interventions; study appraisal and synthesis methods; results; limitations; conclusions and implications of key findings; systematic review registration number. | 1 |
| **INTRODUCTION** | | | |
| Rationale | 3 | Describe the rationale for the review in the context of what is already known. | 1–2 |
| Objectives | 4 | Provide an explicit statement of questions being addressed with reference to participants, interventions, comparisons, outcomes, and study design (PICOS). | 2 |
| **METHODS** | | | |
| Protocol and registration | 5 | Indicate if a review protocol exists, if and where it can be accessed (e.g., Web address), and, if available, provide registration information including registration number. | 2 |
| Eligibility criteria | 6 | Specify study characteristics (e.g., PICOS, length of follow-up) and report characteristics (e.g., years considered, language, publication status) used as criteria for eligibility, giving rationale. | 3 |
| Information sources | 7 | Describe all information sources (e.g., databases with dates of coverage, contact with study authors to identify additional studies) in the search and date last searched. | 3 |
| Search | 8 | Present full electronic search strategy for at least one database, including any limits used, such that it could be repeated. | Supplementary  Material  (Table S2) |
| Study selection | 9 | State the process for selecting studies (i.e., screening, eligibility, included in systematic review, and, if applicable, included in the meta-analysis). | 3 |
| Data collection process | 10 | Describe method of data extraction from reports (e.g., piloted forms, independently, in duplicate) and any processes for obtaining and confirming data from investigators. | 3–4 |
| Data items | 11 | List and define all variables for which data were sought (e.g., PICOS, funding sources) and any assumptions and simplifications made. | 3–4 |
| Risk of bias in individual studies | 12 | Describe methods used for assessing risk of bias of individual studies (including specification of whether this was done at the study or outcome level), and how this information is to be used in any data synthesis. | 4 |
| Summary measures | 13 | State the principal summary measures (e.g., risk ratio, difference in means). | 3 |
| Synthesis of results | 14 | Describe the methods of handling data and combining results of studies, if done, including measures of consistency (e.g., I^2^) for each meta-analysis. | 3–4 |

| **Section/topic** | **Item**  **No.** | **Checklist item** | **Reported on**  **page No.** |
| --- | --- | --- | --- |
| Risk of bias across studies | 15 | Specify any assessment of risk of bias that may affect the cumulative evidence (e.g., publication bias, selective reporting within studies). | 3 |
| Additional analyses | 16 | Describe methods of additional analyses (e.g., sensitivity or subgroup analyses, meta-regression), if done, indicating which were pre-specified. | 4 |
| **RESULTS** | | | |
| Study selection | 17 | Give numbers of studies screened, assessed for eligibility, and included in the review, with reasons for exclusions at each stage, ideally with a flow diagram. | Supplementary  Material  (Figure S1) |
| Study characteristics | 18 | For each study, present characteristics for which data were extracted (e.g., study size, PICOS, follow-up period) and provide the citations. | 4–5 |
| Risk of bias within studies | 19 | Present data on risk of bias of each study and, if available, any outcome level assessment (see item 12). | Supplementary  Material  (Figure S2,  Figure S3) |
| Results of individual studies | 20 | For all outcomes considered (benefits or harms), present, for each study: (a) simple summary data for each intervention group  (b) effect estimates and confidence intervals, ideally with a forest plot. | 5, 10 |
| Synthesis of results | 21 | Present results of each meta-analysis done, including confidence intervals and measures of consistency. | 5, 10 |
| Risk of bias across studies | 22 | Present results of any assessment of risk of bias across studies (see Item 15). | Supplementary  Material  (Figure S2,  Figure S3) |
| Additional analysis | 23 | Give results of additional analyses, if done (e.g., sensitivity or subgroup analyses, meta-regression [see Item 16]). | 6–8, 10–11 |
| **DISCUSSION** | | | |
| Summary of evidence | 24 | Summarize the main findings including the strength of evidence for each main outcome; consider their relevance to key groups (e.g., healthcare providers, users, and policy makers). | 15–16 |
| Limitations | 25 | Discuss limitations at study and outcome level (e.g., risk of bias), and at review-level (e.g., incomplete retrieval of identified research, reporting bias). | 15–16 |
| Conclusions | 26 | Provide a general interpretation of the results in the context of other evidence, and implications for future research. | 15–16 |
| **FUNDING** | | | |
| Funding | 27 | Describe sources of funding for the systematic review and other support (e.g., supply of data); role of funders for the systematic review. | 16 |

*From:* Moher D, Liberati A, Tetzlaff J, Altman DG, The PRISMA Group (2009). Preferred Reporting Items for Systematic Reviews and Meta-Analyses: The PRISMA Statement. PLoS Med 6(6): e1000097. doi:10.1371/journal.pmed1000097

Table S2: Search strategy

| **MEDLINE (via PubMed)** |
| --- |
| We used the two search formulas given below:  (1) "Diabetes Mellitus, Type 2" [MeSH] AND "Dipeptidyl-Peptidase IV Inhibitors" [MeSH] AND "Insulins" [MeSH]  (2) ("Dipeptidyl-Peptidase IV Inhibitors"[Mesh] OR "DPP-4 inhibitor"[All Fields] OR "DPP-4 inhibitors"[All Fields] OR "DPP4 inhibitor"[All Fields] OR "DPP4 inhibitors"[All Fields] OR "DPP-4i"[All Fields] OR "DPP4i"[All Fields] OR "sitagliptin"[All Fields] OR "alogliptin"[All Fields] OR "vildagliptin"[All Fields] OR "saxagliptin"[All Fields] OR "linagliptin"[All Fields] OR "anagliptin"[All Fields] OR "teneligliptin"[All Fields]) AND ("add"[All Fields] OR "added"[All Fields] OR "add-on"[All Fields] OR "adding"[All Fields] OR "additional"[All Fields] OR "addition"[All Fields] OR "combine"[All Fields] OR "combined"[All Fields] OR "combination"[All Fields] OR "add on therapy"[All Fields] OR "co-administration"[All Fields] OR "coadministration"[All Fields]). |
| **EMBASE (via Ovid)** |
| L1: SEA INSULIN? OR LISPRO OR ASPART OR GLULISTINE OR NEURAL OR  ISOPHANE OR GLARGINE OR DETEMIR OR DEGLUDEC  L2: SEA SINGLE? OR MONOTHERAP? OR MONOTREAT? OR MONO OR ALONE  L3: SEA L1(3A)L2  L4: SEA DIPEPTIDYL PEPTIDASE IV INHIBITOR+NT/CT OR SITAGLIPTIN? OR  VILDAGLIPTIN? OR ALOGLIPTIN? OR LINAGLIPTIN? OR TENEGLIPTIN?  OR ANAGLIPTIN? OR SAXAGLIPTIN? OR (DIPEPTIDYL(W)PEPTIDASE(W)(IV  OR 4) OR DPPIV OR DPP4)(2A)INHIBIT?  L5: QUE ADD OR ADDON OR ADD(W)ON OR ADDITIONAL OR COMBIN? OR PLUS  OR ADD ON THERAPY+NT/CT OR DRUG COMBINATION+NT/CT OR CB/CT  L6: SEA RCT OR RANDOM? OR RANDOMIZATION+NT/CT OR RANDOMIZED  CONTROLLED TRIAL+NT/CT  L7: SEA METAANAL? OR META(W)ANALY? OR META ANALYSIS+NT/CT OR  SYSTEM?(2A)REVIEW? OR SYSTEMATIC REVIEW/CT  L8: SEA L3 AND L4 AND L5 AND (L6 OR L7) |
| **Cochrane Library** |
| #1: MeSH descriptor: [Diabetes Mellitus, Type 2] explode all trees  #2: MeSH descriptor: [Dipeptidyl-Peptidase IV Inhibitors] explode all trees  #3: MeSH descriptor: [Insulins] explode all trees  #4: #1 and #2 and #3 |
| **Clinical Trials.gov** |
| "INSULIN" AND ("DIPEPTIDYL PEPTIDASE IV INHIBITORS" OR "DIPEPTIDYL PEPTIDASE-IV INHIBITORS" OR "DIPEPTIDYL PEPTIDASE 4 INHIBITORS" OR "DIPEPTIDYL PEPTIDASE-4 INHIBITORS" OR "DPP-4" OR "DPP4" OR "DPP-IV" OR "DPP 4" OR "SITAGLIPTIN" OR "VILDAGLIPTIN" OR "ALOGLIPTIN" OR "LINAGLIPTIN" OR "TENELIGLIPTIN" OR "ANAGLIPTIN" OR "SAXAGLIPTIN") AND INFLECT EXACT ("Completed" OR "Terminated" OR "Suspended" OR "Withdrawn") [OVERALL-STATUS] AND INFLECT EXACT "Interventional" [STUDY-TYPES] AND INFLECT EXACT ("Adult" OR "Senior") [AGE-GROUP] |
| **Google Scholar** |
| "insulin" AND ("sitagliptin" OR "vildagliptin" OR "alogliptin" OR "linagliptin" OR "teneligliptin" OR "anagliptin" OR "saxagliptin")  For a hand search using Google Scholar, we conducted a broader literature search. Note that we did not search for patents and citations when we used Google Scholar. |

There were no restrictions on publication status, language, or publication date. The literature search began on September 1, 2015, and the last search was conducted in all databases on December 1, 2016.


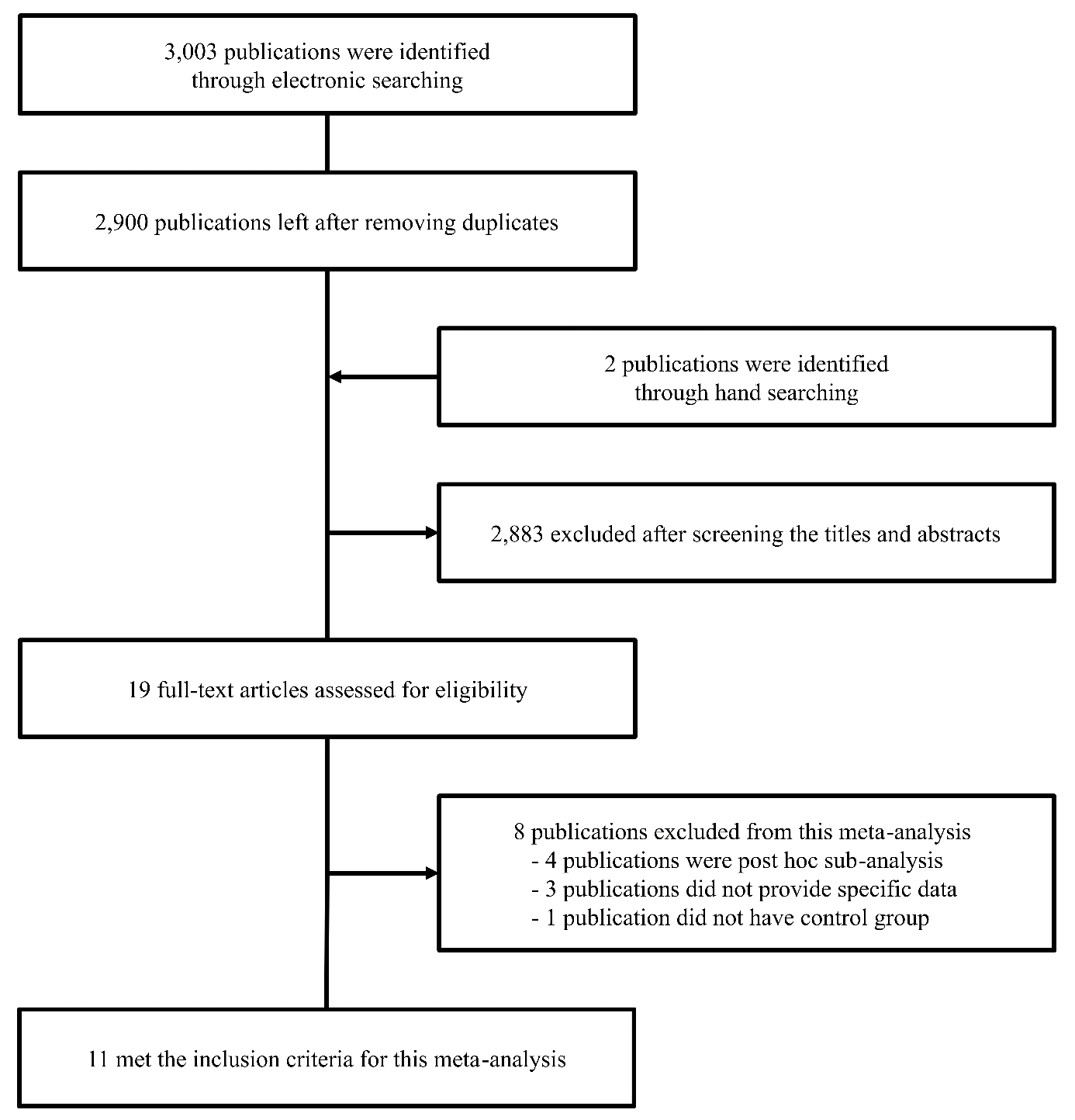


Figure S1: Flowchart for selecting included studies.


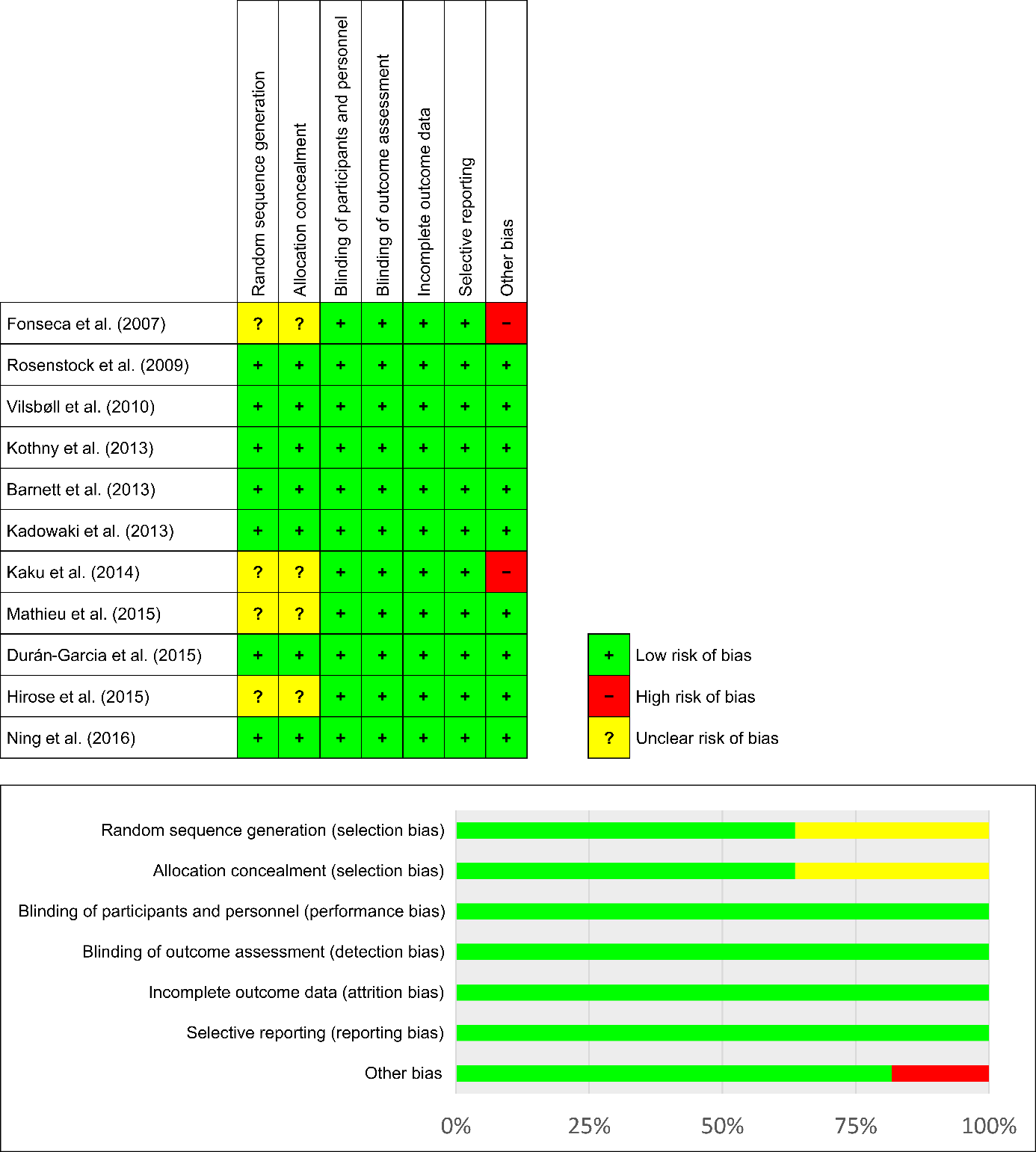


Figure S2: Risk of bias assessment for ΔHbA1c. We evaluated "Other bias" as high risk when the placebo run-in period was not set.


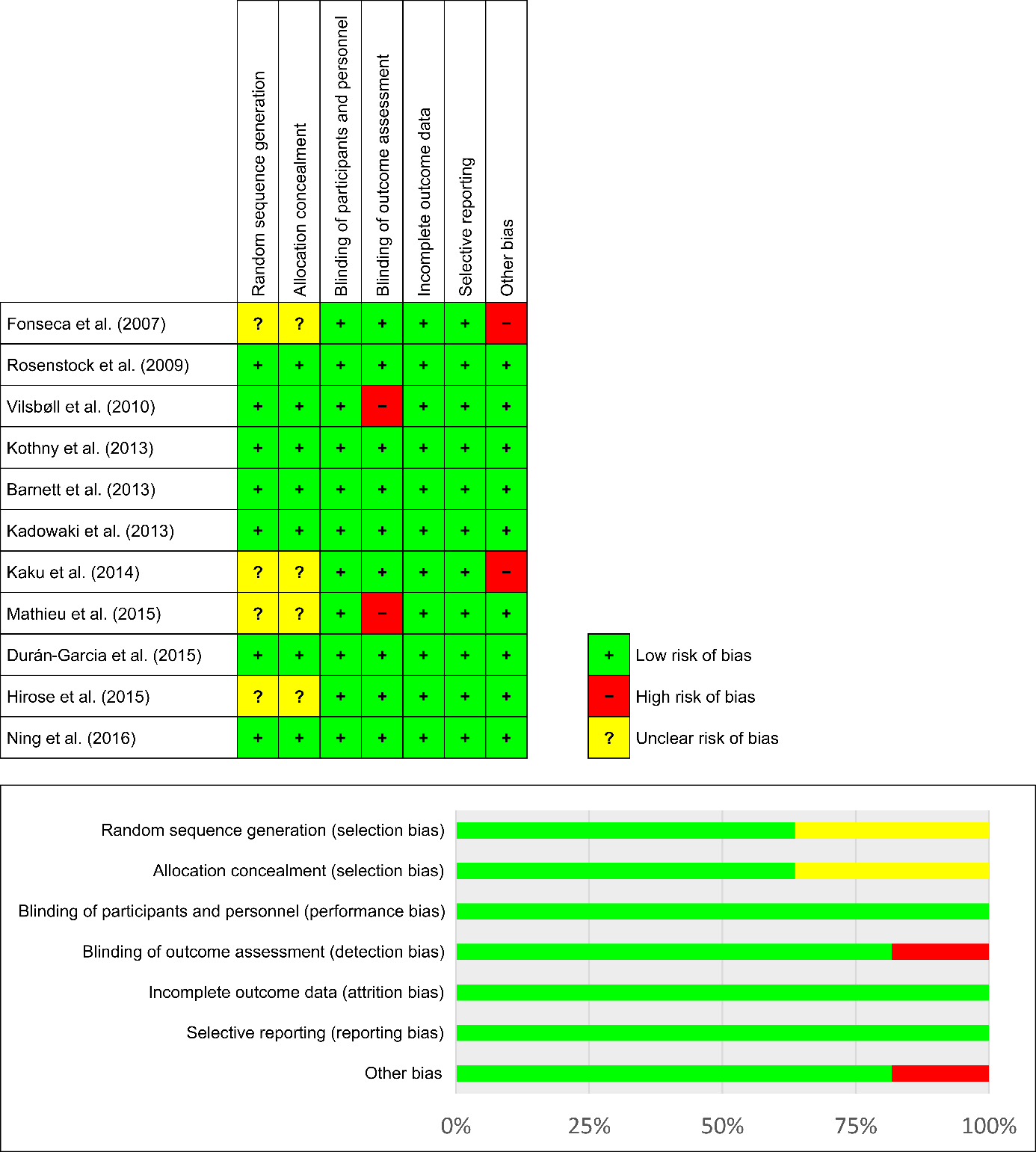


Figure S3: Risk of bias assessment for the incidence of hypoglycemia. We evaluated "Blinding of outcome assessment" as high risk when symptomatic hypoglycemia was adopted as the definition of hypoglycemia.


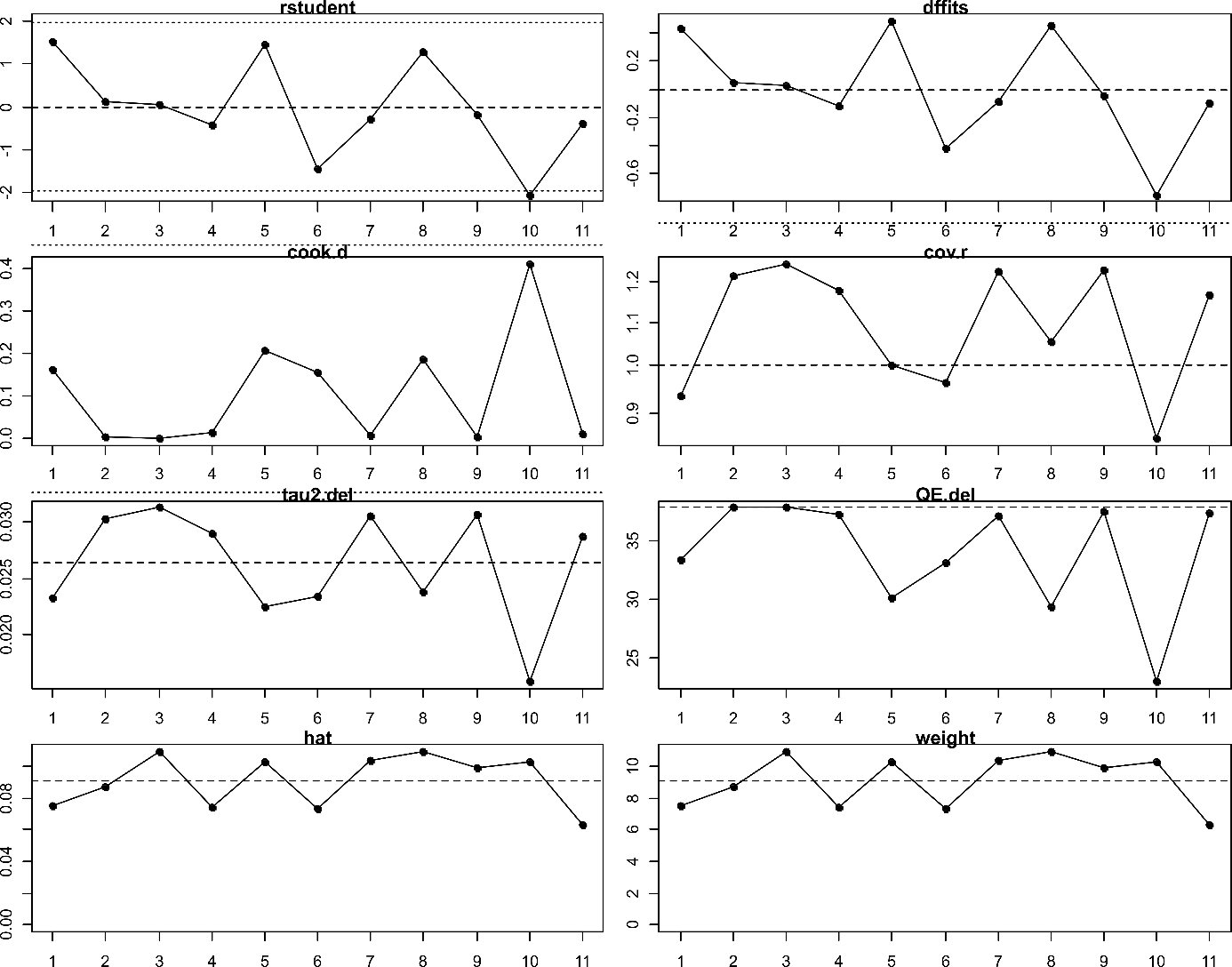


Figure S4: Diagnostic plot of the random-effects model for ΔHbA1c


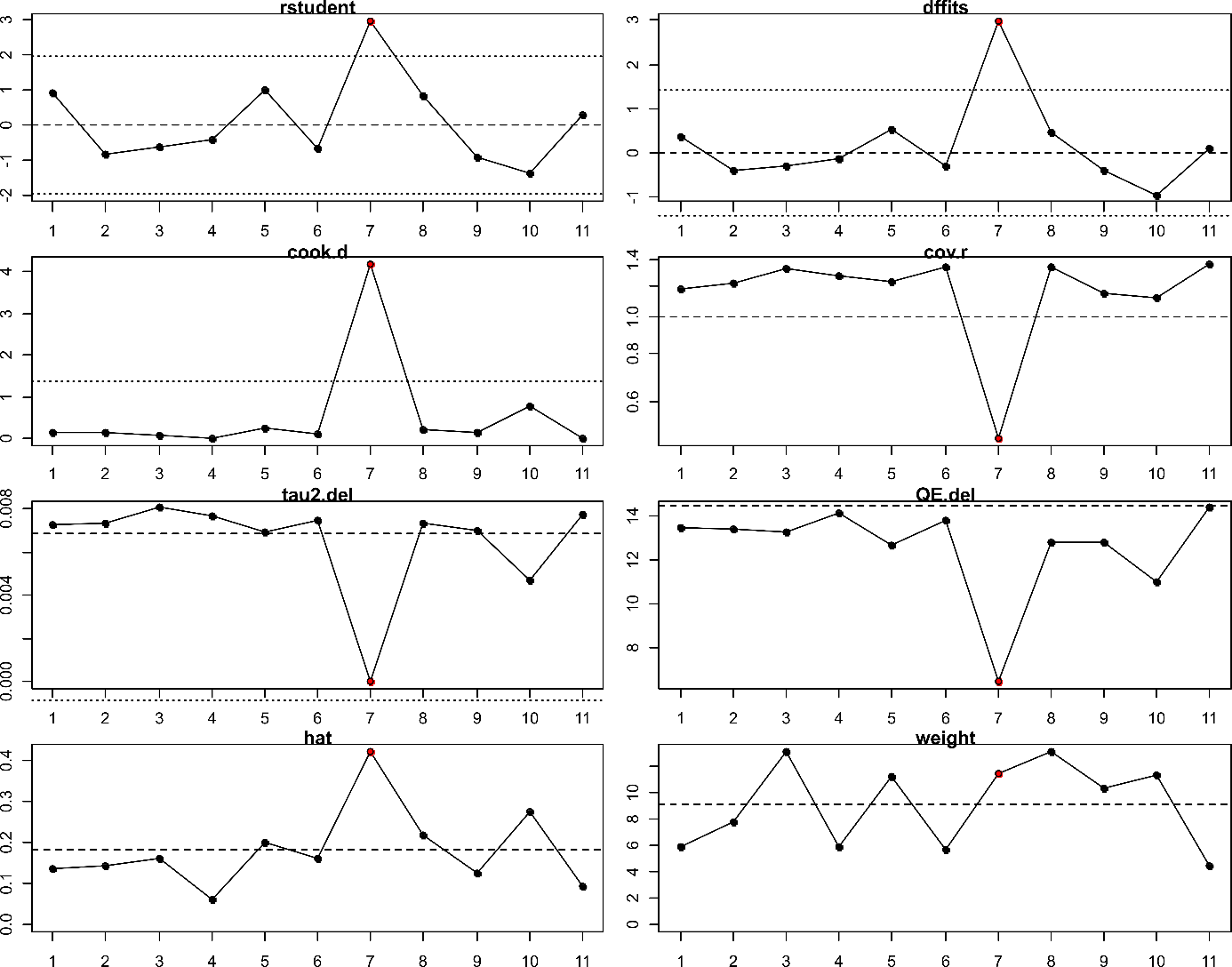


Figure S5: Diagnostic plot of the mixed-effects model for ΔHbA1c. Study 7 is the study by Kaku et al. (2014).


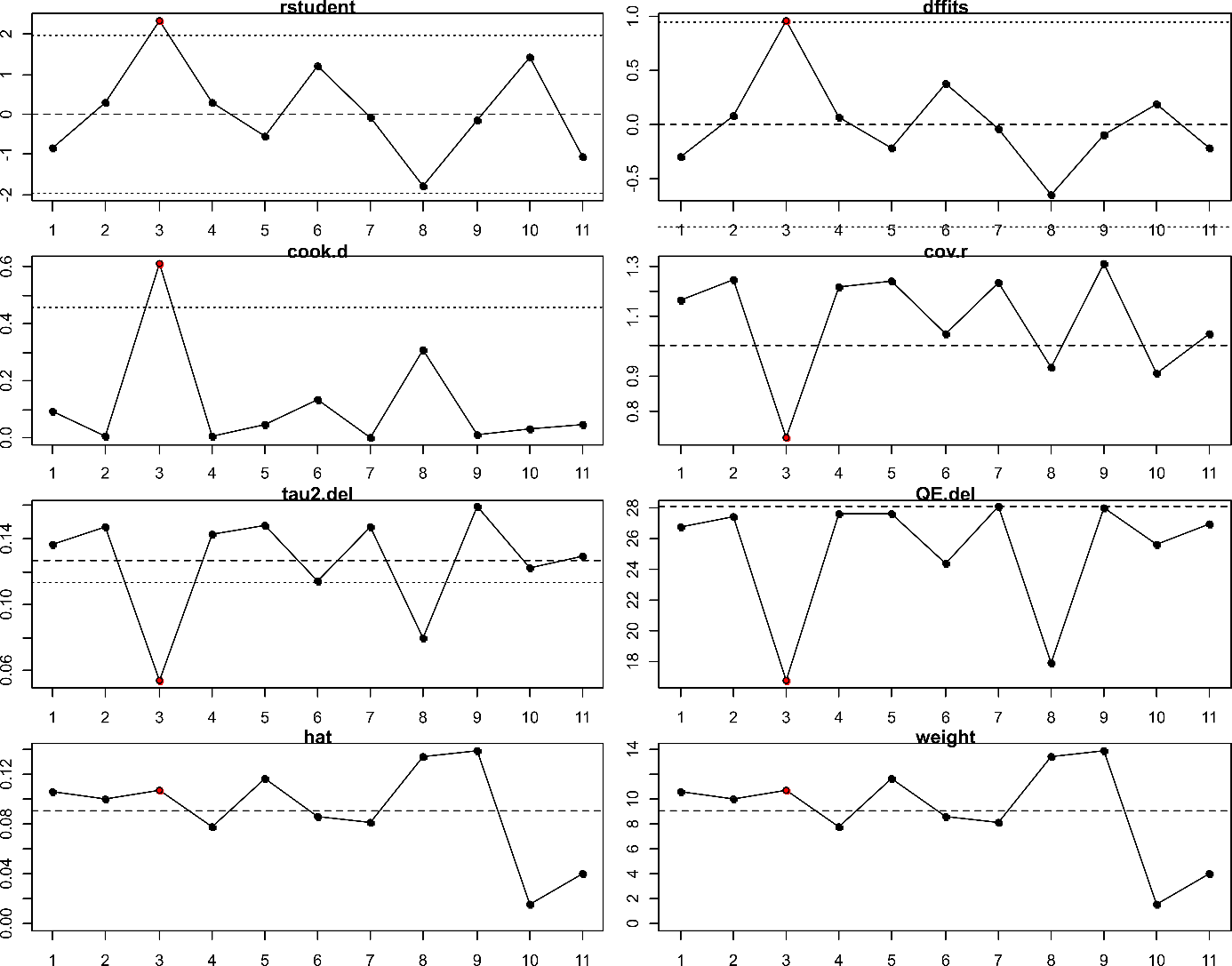


Figure S6: Diagnostic plot of random-effects model for hypoglycemia. Study 3 and study 8 are the studies by Vilsbøll et al. (2010) and Mathieu et al. (2015), respectively.
